# Supplementary figures and images for: Exploration of microbial communities contributing to effective methane production from scum under anaerobic digestion
Source: PLoS One. 2021 Sep 30;16(9):e0257651. doi: 10.1371/journal.pone.0257651 (PMC8483398; doi:10.1371/journal.pone.0257651)

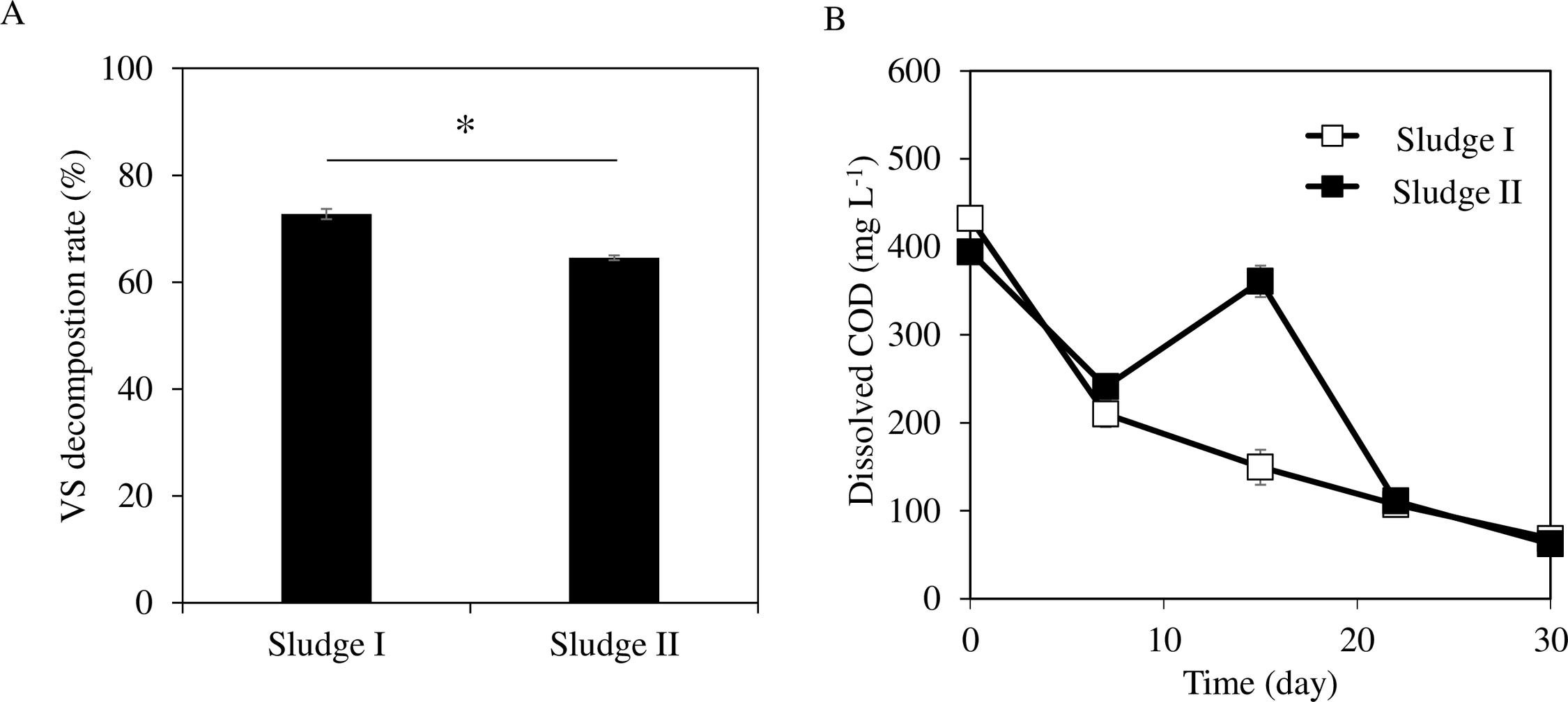

Supplement: S1 Fig — VS decomposition rate (A) and transition of dissolved COD concentration (B). Error bars represent the standard deviation of the mean (n = 3). (TIF) [file pone.0257651.s001.tif]

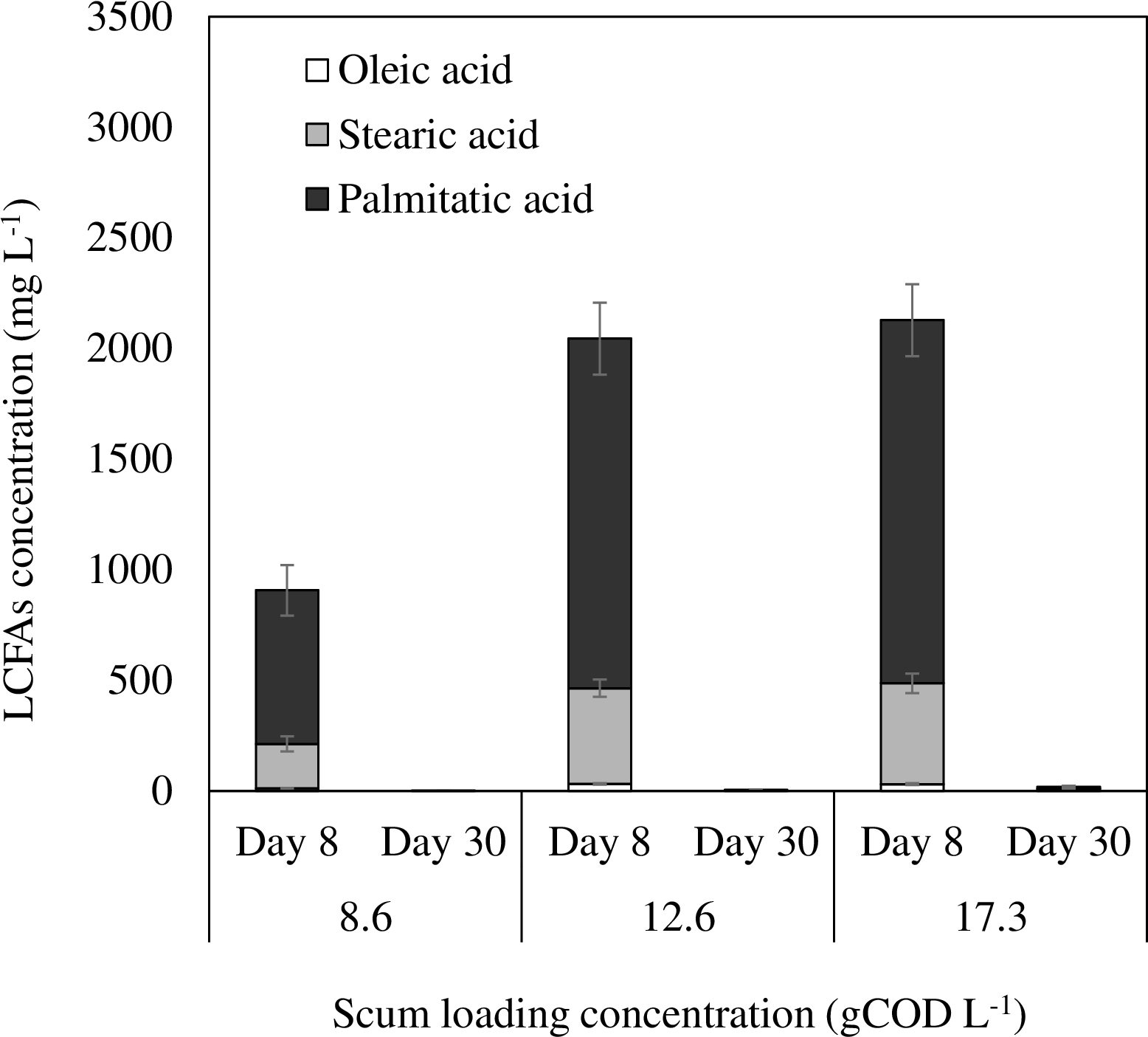

Supplement: S2 Fig — Error bars represent the standard deviation of the mean (n = 3). (TIF) [file pone.0257651.s002.tif]

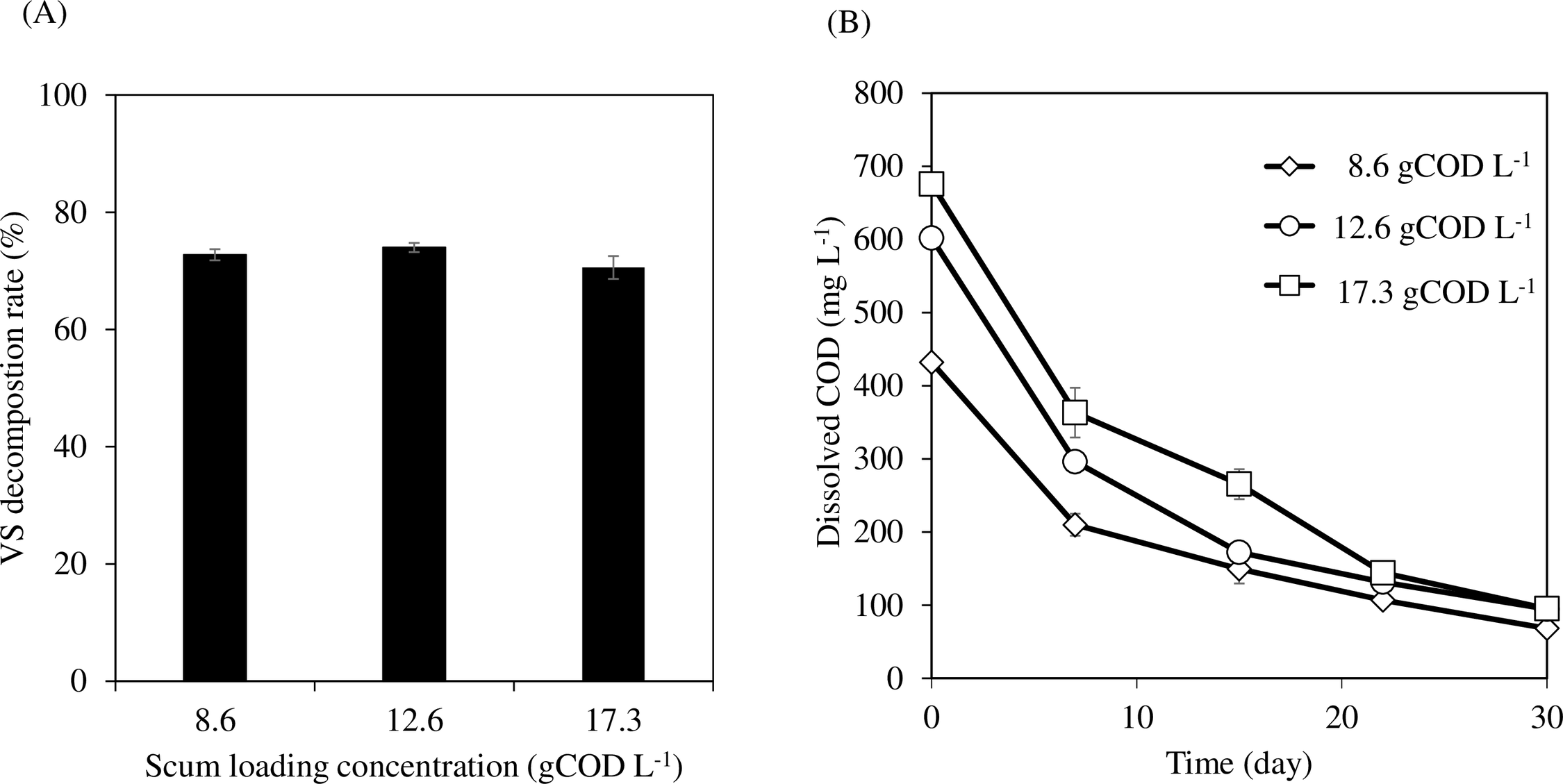

Supplement: S3 Fig — VS decomposition rate (A) and transition of dissolved COD concentration (B) at the different initial scum loading concentrations. Error bars represent the standard deviation of the mean (n = 3). (TIF) [file pone.0257651.s003.tif]

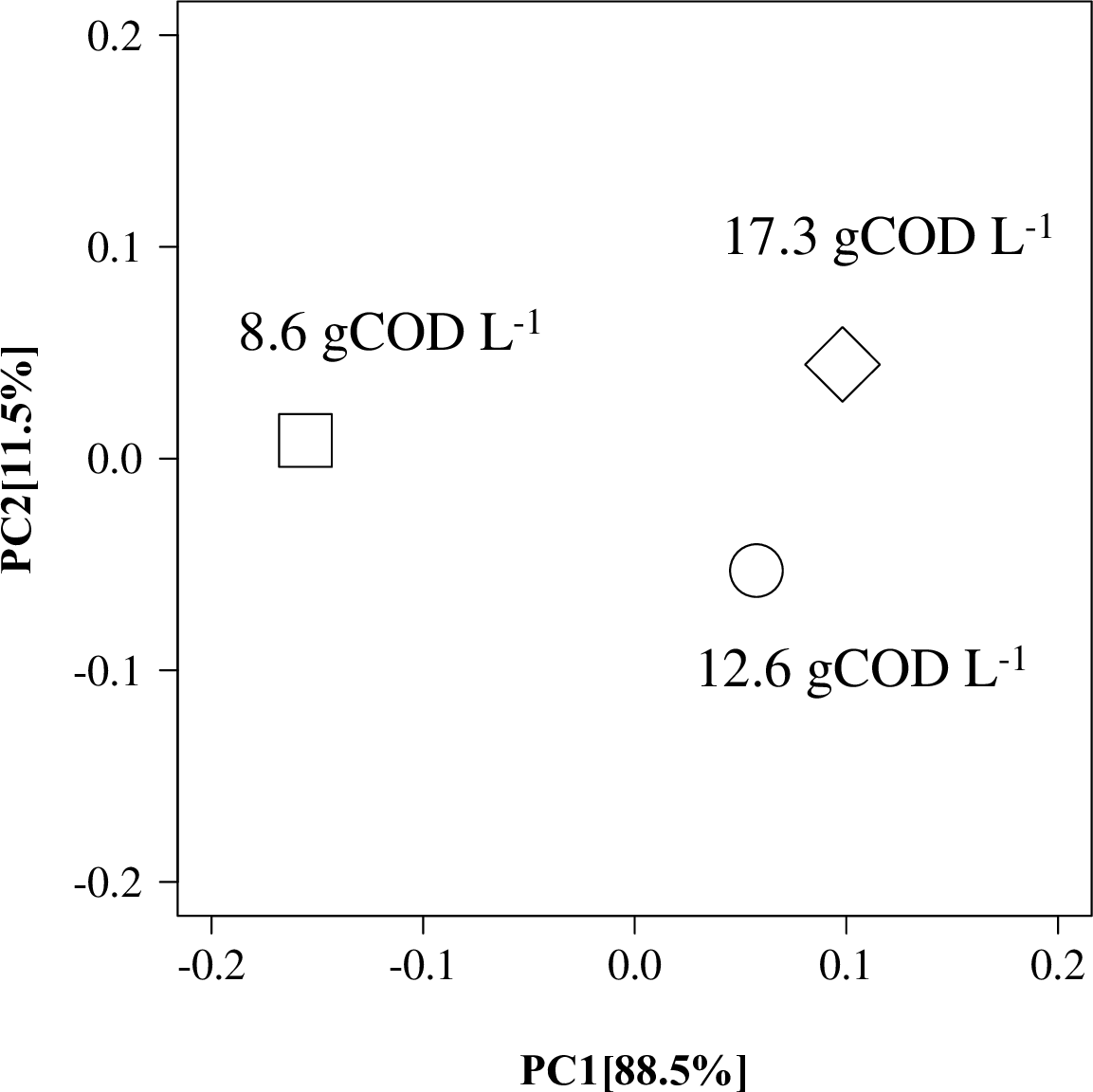

Supplement: S4 Fig — (TIF) [file pone.0257651.s004.tif]

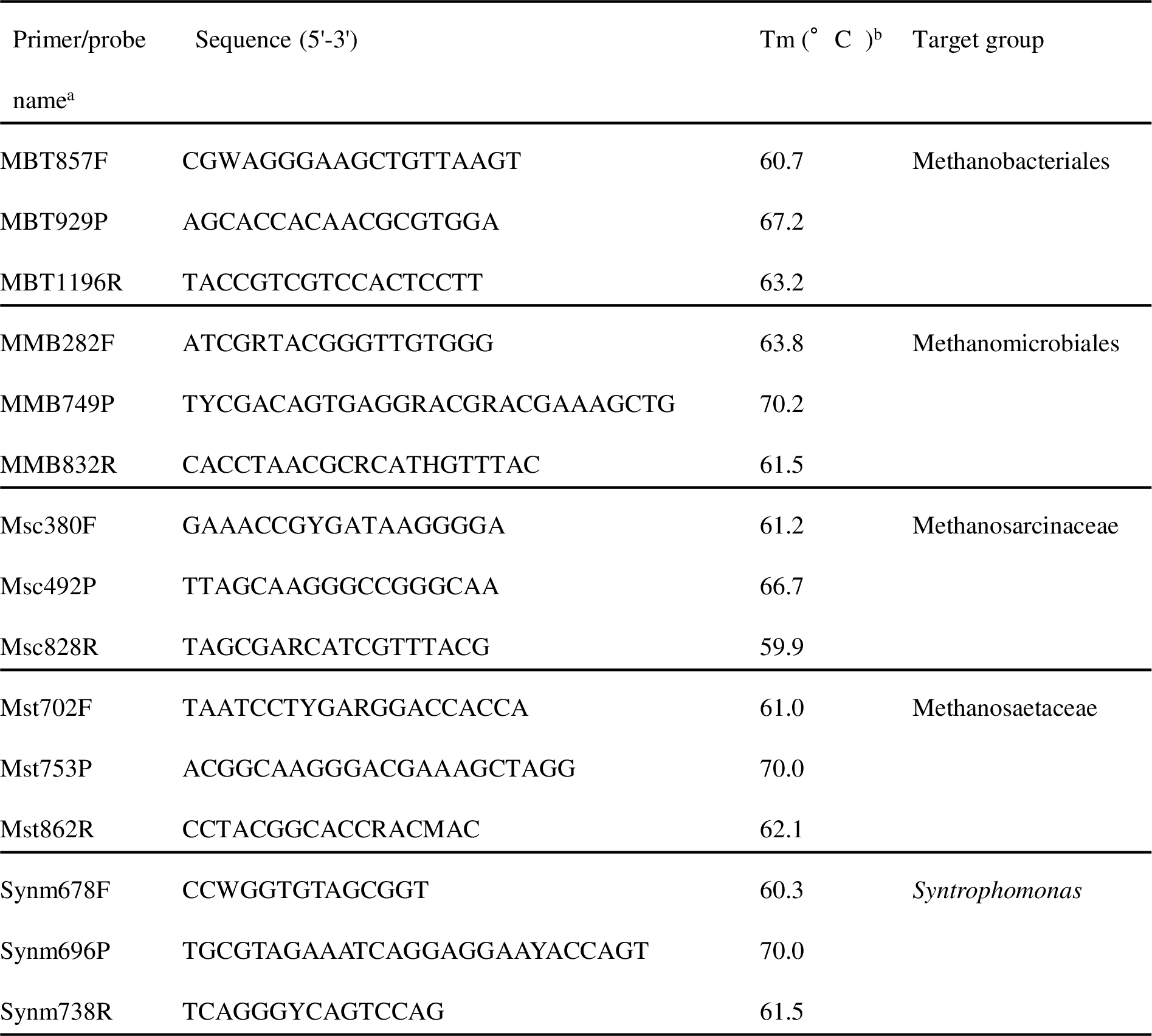

Supplement: S1 Table — a F = forward primer; P = TaqMan probe; R = reverse primer. b Tm of each oligo was cited from its reference article. (TIF) [file pone.0257651.s005.tif]
