# Supplementary material for: Exploration of microbial communities contributing to effective methane production from scum under anaerobic digestion
Source: PLoS One. 2021 Sep 30;16(9):e0257651. doi: 10.1371/journal.pone.0257651 (PMC8483398; doi:10.1371/journal.pone.0257651)
Supplement: Attachment — Submitted filename: PONE-D-21-16045.docx [file pone.0257651.s006.docx]

**PONE-D-21-16045**

The authors documented the microbial community responsible for the scum degradation in anaerobic sludge. This strategy could significantly degrade the scum with a methane production efficiency of 73%. This also aided the degradation of proteins and polysaccharides along with long-chain fatty acids. Changes in microbial community structure clearly showed the potential of adopted sludge and applied strategy for scum degradation. The study may be considered after addressing the comments during revision.

**Comments:**

- Did the study compared with control operation-Consumption of the substrate was confirmed by the cessation of biogas generation. Cessation of biogas cannot be considered the only parameter to judge the influence of scum in the digester.
- Line 233-Considering the low methane production in Sludge II, it was inferred that acetate conversion to methane was inhibited till Day 15
- Did Degradation of oleic acid and palmitic acid observed in this study-if degraded then what is the end product in the digester.
- What is more recommended – i) treatment of scum in physical or chemical process with enhanced methane (>50%) efficiency or ii) biological treatment of scum with lower methane conversion rate of 26 % as shown in this study.
- What could be the non-dissolved and complex substances in the undigested sludge?
