# Supplementary material for: Exploration of microbial communities contributing to effective methane production from scum under anaerobic digestion
Source: PLoS One. 2021 Sep 30;16(9):e0257651. doi: 10.1371/journal.pone.0257651 (PMC8483398; doi:10.1371/journal.pone.0257651)
Supplement: Attachment — Submitted filename: Response to Reviewers.docx [file pone.0257651.s007.docx]

5. Review Comments to the Author

Please use the space provided to explain your answers to the questions above. You may also include additional comments for the author, including concerns about dual publication, research ethics, or publication ethics. (Please upload your review as an attachment if it exceeds 20,000 characters)

Reviewer #1: PONE-D-21-16045

The authors documented the microbial community responsible for the scum degradation in anaerobic sludge. This strategy could significantly degrade the scum with a methane production efficiency of 73%. This also aided the degradation of proteins and polysaccharides along with long-chain fatty acids. Changes in microbial community structure clearly showed the potential of adopted sludge and applied strategy for scum degradation. The study may be considered after addressing the comments during revision.

Reply: Thank you for your comments.

• Did the study compared with control operation-Consumption of the substrate was confirmed by the cessation of biogas generation. Cessation of biogas cannot be considered the only parameter to judge the influence of scum in the digester.
Reply: We assumed that this comment refers to the description in line 101. We checked the gas-cessation before the batch experiments to avoid introducing substrates which were used in the pre-incubation period. We did not consider the cessation of biogas as a parameter to judge the influence of scum in the digester.

•Line 233-Considering the low methane production in Sludge II, it was inferred that acetate conversion to methane was inhibited till Day 15
Reply: Given that your comment does not appear to contain any criticism or question, we did not change anything in the manuscript in this respect.
• Did Degradation of oleic acid and palmitic acid observed in this study-if degraded then what is the end product in the digester.
Reply: Oleic acid and palmitic acid were oxidized to VFAs (acetate or propionate). Then they are converted to methane. We added the following. “LCFAs were degraded to VFAs (line 59 in the revised manuscript).”
• What is more recommended – i) treatment of scum in physical or chemical process with enhanced methane (>50%) efficiency or ii) biological treatment of scum with lower methane conversion rate of 26 % as shown in this study.
Reply: The aim of this study was to reveal the cardinal microbes mainly contributing to scum degradation. Our microbiological findings (summarized in lines 434–440) will provide new possibilities to enhance scum degradation. For example, if these microbes are more enriched, a higher methane conversion rate can be achieved. The treatment of scum in a physical or chemical process is certainly effective, but will involve higher costs than biological treatment. In addition, biological treatment can be combined with physical or chemical treatment.
• What could be the non-dissolved and complex substances in the undigested sludge?

Reply: Experiment II showed that protein- or polysaccharide-hydrolyzing bacteria were enriched with respect to the scum loading rate. It was suggested that these bacteria played an important role during scum degradation. Because LCFAs were degraded almost completely, proteins and polysaccharides seemed to remain undigested in Sludge II.

We revised the sentence to “These results suggested that non-dissolved, complex substances, such as proteins or polysaccharides, remained undigested in Sludge II (lines 235–237).”

Reviewer #2: The present manuscript deals with the analysis of the microbial communities which are involved during batch anaerobic digestion of rich-in oils and grease, scum. The study comes into the aims and scopes of the journal and presents a detail analysis of the microbial community, however there are some points that should be addressed, before publication.

The novelty of the study is not presented clearly. In addition, some measurements are missing. Moreover, the authors should have performed blank experiments (only inoculum of sludge 1 and sludge 2 without scum) the methane of which should have been subtracted by the methane of the scum and also microbial analysis should have been performed in that experiments so as direct comparison with the microbial identification of scum, should have been conducted.

Reply: Thank you for your comments.

1) The authors should be careful with the English writing and diction and the whole presentation of their manuscript. There are so many grammatical and syntax errors.

Reply: We appreciate that you pointed out these errors, which we corrected throughout the manuscript.
2) The abstract needs revision to be more clear

Reply: We revised the abstract according to your suggestion.
3) The authors use first plural form in order to describe what they have done. To my opinion, is better to present the methodology and results in third singular

Reply: We appreciate your suggestion. Descriptions were revised using third-person singular.
4) Line 64: disposal of scum does not lead to biomass washout. Please revise

Scum is formed by the adsorption of floating substrates, such as long-chain fatty acids (LCFAs), to the surface of biomass. We used the word “biomass” to indicate organic waste, which is the substrate for anaerobic digestion. This is why a disposal of scum leads to the washout of organic waste. we revised the word “biomass” to “organic waste” (line 57 in the revised manuscript)

5) Lines 66-67, 71-71, line 74, lines 228-229: syntax problems

Reply: We revised the respective sentences as follows.

“LCFAs, which are the main cause of the scum formation, are produced by hydrolysis of lipids, and they have some embarrassing features” to “The main cause of scum formation is LCFAs, which are produced by the hydrolysis of lipids” (lines 58–59)”.

“decreased methane generation by more than 50%” to “reduced methane generation by more than 50% (line 63)”.

“The biomass washout by removing the accumulated scum has impaired the advantage of lipids severely” to “Biomass washout severely impairs the high methane convertibility of lipids (lines 66)”.

“In Sludge I, VFAs concentration then decreased gradually, and they became undetectable on Day 22” to “In Sludge I, VFA concentrations then decreased gradually, and became undetectable on Day 22 (Lines 213–214)”.
6) Line 86: grammatical errors

Reply: We revised the sentence as follows:

“Ziels et al., (2016) showed that acclimation with waste restaurant oil increased the relative abundance of genus *Syntrophomonas*, which has a capability degrading various fatty acids”

to “Ziels et al., (2016) showed that acclimation with waste restaurant oil increased the relative abundance of the genus *Syntrophomonas*, which has the capability of degrading various fatty acids (lines 76–78)”.
7) Lines 91-94: not well written. not apparent the innovation. The authors should give the research gap that their work fills in.

Reply: We revised the sentence as follows:

“Microbiological findings on anaerobic scum digestion will enable new approaches to solve the problems caused by scum. In this study, we aimed to reveal the microbes contributing to an effective scum degradation. We pre-incubated a collected sludge with scum to enrich scum-degrading microbes. Additionally, to investigate the core scum-degrading microbes in this pre-incubated sludge, we evaluated the changes in microbial communities according to various loading rates (lines 82–86).”

8) line 108: which are the characteristics of sludge in terms of COD, pH TS and VS? please give a table

Reply: We added a table on the respective parameters (Table 1, line 114). We did not measure pH.
9) line 108: Was there any protocol that was followed?

Reply: There were no protocols followed. Since scum contains LCFAs, we aimed to adapt the microorganisms to LCFAs (olive oil) first.
10) which was the reason of developing the sludge 2? Please comment.

Reply: Sludge II was incubated with glucose to avoid the starvation of microorganisms, not to adapt them to glucose. Glucose is a fundamental nutrient contained in food waste. We added the words “to avoid starvation” (lines 101–102).

11) Line 115: experiments, line 117: better 40 mL

Reply: We revised the words (line 106, 107)
12) which was the protocol followed for the batch methane experiments? Please provide a table with the characteristics of scum and sludge

Reply: We added a table (Table 1, line 114).
13) the authors should have performed blank and control experiments.

For the following reasons, we designed the experimental conditions without typical blank/control lines.

1) A previous study considered that cessation of biogas indicates the substrates were consumed almost completely (Baba et al., 2013). Since we used sludges for which gas-cessation was confirmed, we assumed that it was not necessary to perform blank experiments.

2) A control experiments, we could have performed batch tests using a sludge before pre-incubation with scum. However, the long-term storage of scum may change its characteristics. It was, thus, impossible to prepare the exact same scum or sludge. Since our main purpose was to explore the microbes, we assessed the ability of Sludge I in Experiment 1 by comparison with a sludge of different origin.

Baba, Y., Tada, C., Fukuda, Y., Nakai, Y. Improvement of methane production from waste paper by pretreatment with rumen fluid. Bioresour. Technol. 2013. 128, 94–99. <https://doi.org/10.1016/j.biortech.2012.09.077>

14) Lines 129-131: not clear

Reply: We revised the sentences to ‘The batch experiments were conducted in sextuplicate lines until Day 8, and three of these replicates were opened on Day 8 for DNA extraction and LCFA analysis. Then the batch experiments were continued using the remaining triplicates’ (lines 108–112).
15) Line 216, 218: methane production of sludge

Reply: We revised the sentences (lines 204, 205)
16) Line 248: units?

Reply: We added the units (mg L^-1^) (line 234)
17) Lines 252-253: repeat

Reply: The sentence was deleted.
18) Line 253: enzymes

Reply: We revised the word (line 239).
19) Table 1: units? line 299: units?

Reply: We revised Table 1 to a figure (Fig 3) and units were added.

20) Line 309 :syntax

Reply: We deleted the words ‘Methanomicrobiales Methanobacteriales’
21) Lines 395-305: syntax errors and repeat

Reply: We revised the passage as following:

“After quality filtering, denoising, and merging paired-end amplicon reads, 48,529–52,393 reads (average 49,957) were obtained from the raw MiSeq data.” to “48,529–52,393 non-chimeric reads (average 49,957) were obtained from the raw MiSeq data (lines 354-355).

22) Line 405: bacteria , line 411? viales?

Revised: We revised the words (line 364, 370)

Reviewer #3: The present study aimed to investigate the biomethane productivity of scum waste along with changes in microbial communities with respect to various loading rates. Firstly, enrichment of scum-degrading microbes was evaluated with the collected sludge and scum digestibility was assessed by comparing with another collected sludge. The results showed 1.3 times higher methane conversion rate (73%) and faster LCFA degradation in the pre-incubated sludge with scum. Secondly, the scum loading rates' influence on the microbial community was studied, wherein the increased 16S rRNA copy numbers for the syntrophic fatty-acid degrader, Syntrophomonas, and hydrogenotrophic methanogen were observed in scum high-loaded samples. These results indicated profiles of microorganisms that degrade LCFAs in scum anaerobic digestion. The objective of the study is novel, but the methodology representation and results discussion are not appropriate. Recommending for major revision and below suggested comments could be useful to improve the manuscript quality.

Reply: Thank you for your comments.

• The abstract is too lengthy and contains general discussion, recommending modify the abstract as per the specific objectives of the study along with including the obtained results.

Reply: We revised the abstract.
• Maintain a similar font size throughout the manuscript and give the appropriate numbering to the sections.

Reply: We checked and revised the text entirety. We appreciate your careful reading.
• The compositional details of scum such as VS, TS, Total COD, Soluble COD, VFA, and LCFA are is not included.

Reply: We added a table on VS and TS, and the total COD of scum or sludges (Table 1, line 114). Because previous studies referred to here (Young et al., 2013, 2016) did not show VFA and LCFA in scum, this was not analyzed. We believe VS and TS, and the total COD are adequate.

Young, B., Delatolla, R., Kennedy, K. Biodegradability and mesophilic co-digestion of municipal sludge and scum. Bioprocess Biosyst. Eng. 2013. 36, 1703–1714. <https://doi.org/10.1007/s00449-013-0945-z>

Young, B., Kennedy, K., Delatolla, R., Sherif, R.. Semi-continuous mesophilic anaerobic co-digestion of thermally pretreated scum. Water Qual. Res. J. Canada 2016. 51, 117–127. <https://doi.org/10.2166/wqrjc.2016.024>

• The inoculum loading rates in Experiment 1 and 2 are not included.

Reply: 40 ml of the inoculum (Sludge I or Sludge II) was loaded in each experiment (lines 106-107, line 122).
• The methodology is not clear, the authors were chosen different sludges (Sludge I and II), loading rates, and different batch studies. Suggesting to rewrite the methodology by maintaining the flow also includes a schematic representation of work for better understanding to the readers.

Reply: We added a schematic representation as Figure 1 and revised the methodology (Experiment 1: Assessment of the digestibility of scum, Experiment 2: Exploration of core scum-digesting microbes).
• Important results like VS or COD reduction is missing with respect to operation day.

Reply: Since LCFAs adsorb onto biomass and form flocs, it is difficult to analyze total COD or VS reduction with respect to operation day precisely. Thus, also some previous studies investigating LCFA-degrading microbes did not conduct these analyses (Kurade et al., 2020, Ziels et al., 2016). The results for dissolved COD reduction were added (Figure S3).

Kurade, M.B., Saha, S., Kim, J.R., Roh, H.S., Jeon, B.H. Microbial community acclimatization for enhancement in the methane productivity of anaerobic co-digestion of fats, oil, and grease. Bioresour. Technol. 2020. 296, 122294, https://doi.org/[10.1016/j.biortech.2019.122294](https://doi.org/10.1016/j.biortech.2019.122294)

Ziels, R.M., Karlsson, A., Beck, D.A.C., Ejlertsson, J., Yekta, S.S., Bjorn, A., Stensel, H.D., Svensson, B.H. Microbial community adaptation influences long-chain fatty acid conversion during anaerobic codigestion of fats, oils, and grease with municipal sludge. Water Res. 2016. 103, 372–382. <https://doi.org/10.1016/j.watres.2016.07.043>

• The LCFA utilization with respect to operation day in figure format could be useful rather than a table.

Reply: We revised Table 1 and Table S1 to figure format.
• In table 1, the details of AS 8.6 and CS 8.6 are not provided, suggesting to expand such abbreviations at the bottom of the table.

Reply: We made mistakes. AS 8.6 and CS 8.6 means Sludge I and Sludge II, respectively. We revised the text accordingly.
• The discussed results of scum loading concentration influence on methane productivity is too short and have less discussion, suggesting to elaborate the discussion by covering the influence of various loading rates along with the LCFA utilization with respect to operation day.

Reply: We added a discussion on the influence of loading rates and the inhibitory effect of LCFAs.
• Overall, manuscript quality is very poor and needs significant changes with abstract, methodology and results discussion specifically with VS, LCFA utilization. Some of the supplementary figures could be included in the main content with an appropriate discussion.

Reply: We appreciate your advice. We modified the abstract and methodology for clarity. We included the VFA concentrations (Experiment 2), and the results for dissolved COD reduction were added (Figure S3). Using these results, the effect of scum loading concentrations on methane productivity, along with LCFA utilization, was discussed.
